# Predicting the response of multiple myeloma to the proteasome inhibitor Bortezomib by evaluation of the unfolded protein response

**DOI:** 10.1038/bcj.2016.40

**Published:** 2016-06-10

**Authors:** N Nikesitch, C Tao, K Lai, M Killingsworth, S Bae, M Wang, S Harrison, T L Roberts, S C W Ling

**Affiliations:** 1 Ingham Institute of Applied Medical Research, SWS Clinical School, Western Sydney University, Campbelltown, New South Wales, Australia; 2 Haematology Research Group, SWS Clinical School, University of NSW, Western Sydney University, Ingham Institute of Applied Medical Research, Liverpool, New South Wales, Australia; 3 Department of Haematology, Sydney South West Pathology Service, NSW Health Pathology, Liverpool Hospital, Liverpool, New South Wales, Australia; 4 Anatomical Pathology NSWHP, Liverpool Hospital, Liverpool, New South Wales, Australia; 5 Cancer Pathology and Cell Biology, Ingham Institute of Applied Medical Research, Liverpool, New South Wales, Australia; 6 Flow Cytometry Core Facility, The Westmead Institute for Medical Research, Westmead, New South Wales, Australia; 7 Department of Cancer Medicine, Peter MacCallum Cancer Centre, Melbourne, Victoria, Australia; 8 Sir Peter MacCallum Department of Oncology, University of Melbourne, Parkville, Victoria, Australia; 9 School of Medicine, SWS Clinical School, University of New South Wales, Kensington, New South Wales, Australia; 10 University of Queensland Centre for Clinical Research, Herston, Queensland, Australia; 11 Medical Oncology, SWS Clinical School, Ingham Institute of Applied Medical Research, Liverpool, New South Wales, Australia

Multiple myeloma (MM) remains a predominantly incurable malignancy despite high-dose chemotherapy, autologous stem cell transplant and novel agents.^1^ Proteasome inhibitors (PI) such as Bortezomib have increased the response rate and survival of patients with MM. The overall patient response rate of newly diagnosed MM to Bortezomib and Dexamethasone is about 67%.^2^ In relapsed refractory MM, the response rate is reduced to about 40–60%.^3, 4^ Therefore, there are a significant number of MM patients who are resistant to Bortezomib. Combining Bortezomib with another class of novel drugs, for example, immunomodulatory drugs (IMIDs), such as Lenalidomide, is associated with increased overall response rate of 94% in newly diagnosed myeloma patients^5^ and 64% in relapsed or refractory myeloma.^6^ Although the combination of a proteasome inhibitor and an IMID may yield an improved response rate, it is not always possible due to the cost, availability, local regulatory policies, side-effects profile, convenience and personal preference. Therefore the choice of novel agents (PI or IMID) is predominantly empirical and based on other factors such as side effects and tolerability, making it difficult to choose the best therapy. Currently there is no way of predetermining if a patient will respond to Bortezomib treatment. However, previous studies have shown that XBP1, a key regulator of the unfolded protein response (UPR), predicts sensitivity to Bortezomib, and its level correlates proportionally with sensitivity to Bortezomib.^4^ We therefore aimed to assess if the sensitivity to Bortezomib is dependent on the UPR, and that the expression level of ATF6 mRNA and the size of the endoplasmic reticulum can predict sensitivity to the drug.

ATF6 is a regulator of the UPR and is capable of activating XBP1,^7^ which is a regulator of the UPR and correlates with Bortezomib sensitivity.^4^ Previous studies have shown that the protein expression of ATF6 is reduced in MM cell lines resistant to Bortezomib compared with their parent cell line.^4^ We therefore analysed ATF6 gene expression in Bortezomib sensitive and resistant KMS11 cells (Supplementary Information). Our results showed that ATF6 gene expression decreased with increasing Bortezomib resistance. KMS11 cells resistant to Bortezomib were seen to have substantially lower ATF6 mRNA levels compared with parent sensitive cells (Figures 1a, *P*=0.06), resembling the same trend as seen in protein expression.^4^ These results were also seen in 45 MM patients with various levels of resistance (Supplementary Information). Patient responses after completion of cycle 2 of therapy with Bortezomib were categorized according to the International Myeloma Working Group (IMWG) uniform response criteria.^8^ Complete response (CR), very good partial response (VGPR) or partial response (PR) patients had significantly higher levels of ATF6 mRNA compared with patients with stable disease (SD) or of progressive disease (PD; Figure 1b; *P*=0.007). The mean ATF6 expression of the CR, VGPR and PR patient groups were 3.92-fold higher compared with patients of SD and PD groups. On an individual group basis, there was no significant difference between each group, however there was a significant difference between PR patients vs SD+PD patients (Figure 1b; *P*=0.01). Nonetheless, gene expression levels of ATF6 in SD+PD patients were significantly lower than those seen in patients with CR+VGPR+PR to Bortezomib. Therefore, ATF6 mRNA, a regulator of the UPR is predictive of sensitivity to Bortezomib *in vitro* and in patients.

Expansion of the ER is an important aspect of the UPR when dealing with ER stressed caused by increases in unfolded/misfolded protein. This morphological change assists the UPR by accommodating the flux in protein levels. This has been demonstrated within secretory cells, which have been seen to undergo ER expansion for the production and secretion of large protein quantities.^9^ Therefore, we next examined ER morphology within KMS11-sensitive and -resistant cells, to determine the importance and activity of the UPR in Bortezomib resistance. We first assessed ER morphology by live imaging of sensitive and resistant KMS11 cells using an ER tracker dye and a BioStation IM-Q Time Lapse Imaging System. Comparing the mean fluorescent intensity of the ER in KMS11-resistant cells against KMS11-sensitive cells, there was a 1.35-fold decrease in size (Figures 2a and b; *P*=0.02352; Supplementary Information). ER sizes were larger in sensitive cells, compared with the resistant cells, indicating a higher level of UPR activity. The range of fluorescent measurements from sensitive cells were also seen to be more widely distributed (range of 144 037 CTCF/U) in comparison with resistant cells, which showed a tighter range in fluorescence (44 856 CTCF/U). This is likely a result of Bortezomib-sensitive cells having a highly functional UPR pathway, while Bortezomib-resistant cells have an under functioning or compromised UPR.

To further assess the size of the ER in Bortezomib sensitive and resistant cells, we measured the rough ER (RER) lumen by electron microscopy (see Supplementary Information). At a 40 000 × magnification, up to 10 images were captured of the RER within Bortezomib sensitive and resistant KMS11 cells. The RER lumen of each cell was measured at the 4 widest points, totaling 40 measurements. The RER lumen within the resistant cells was seen to be significantly smaller than those of the sensitive cells (Figures 2c and d; *P⩽*0.0001), with the mean RER lumen width of resistant cells being 10 nm smaller than the mean lumen width of the sensitive cells. The RER measurements of the Bortezomib-sensitive cells were more broadly distributed (range of 36 nm), with the range being substantially greater than those seen in the resistant cells (range of 23 nm). Therefore, the size of the ER is predictive of sensitivity to Bortezomib.

We conclude that UPR activity and function, as measured by ATF6 expression and the size of the ER is reduced in Bortezomib resistance. Further studies are required to determine whether ATF6 expression and ER size are useful predictors of Bortezomib sensitivity and resistance in the clinical setting. Our findings suggest that reduced UPR activity may mediate Bortezomib resistance.

## Figures and Tables

**Figure 1:**
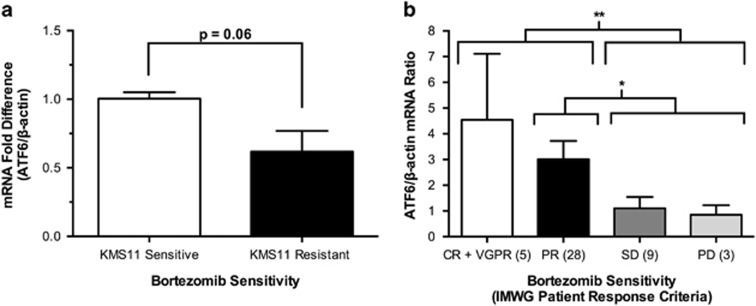
Real-time PCR quantification of ATF6 mRNA expression in a Bortezomib-sensitive and -resistant cell line model and multiple myeloma (MM) patient samples. (**a**) Reduced ATF6 mRNA expression in KMS11 cells resistant (black bar) to Bortezomib relative to the control Bortezomib-sensitive cells (white bar). Data are shown as mean±s.e.m. (*n*=5; *P*=0.06, *t*-test). (**b**) ATF6 mRNA expression in MM patients with increasing Bortezomib resistance according to the IMWG Patient Response Criteria. Complete response (CR) and very good partial response (VGPR) patients (*n*=5) are the most sensitive to Bortezomib, followed by partial response (PR) patients (*n*=28). Patients with stable disease (SD; *n*=9) or progressive disease (PD; *n*=3) were the most resistant to Bortezomib. ATF6 mRNA levels decreased with increasing bortezomib resistance in MM patients. Data are shown as mean values±s.e.m. (*n*=45). Statistical analysis was performed on sensitive patients (groups CR+VGPR and PR) vs resistant patients (groups SD and PD; **P=0.007, *t-*test). There is a statistical difference between PR (*n*=28) and SD+PD patients (*n*=12; *P=0.0107; *t-*test).

**Figure 2:**
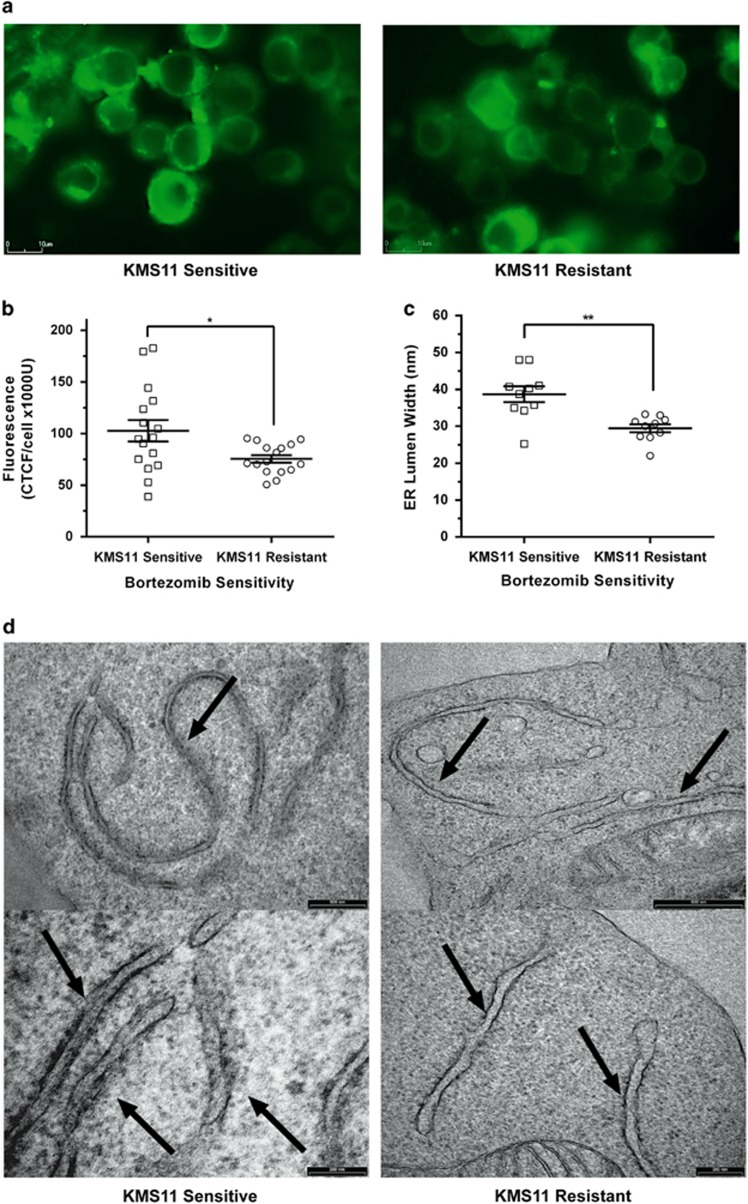
ER imaging of Bortezomib-sensitive and -resistant KMS11 cells. (**a**) Representative images of live cell staining of KMS11-sensitive and -resistant cells, incubated with 100 nm of ER tracker dye (green). Images were captured at × 80 magnification. (**b**) ER fluorescence/area of KMS11-sensitive (*n*=16) and -resistant (*n*=16) cells using an ER tracker dye. Data are shown as mean of values (centre bar)±s.e.m. (error bars; *n*=16; *P=0.0235, *t-*test). Scale bar, 10 μm. Dot plots of individual data values. (**c**) Mean RER lumen widths of KMS11-sensitive cells (*n*=10) and KMS11-resistant cells (*n*=10) at × 40 000 magnification as measured by electron microscopy. Data are shown as mean measurement values±s.e.m. (**P=<0.0001, *t* test) (bar and error bar). Each dot plot point represents the average of the 4 broadest points measured for each RER lumen (**d**) Electron microscopy of the ER lumen (shown by arrows) in KMS11 Bortezomib sensitive (left panels) and resistant cells (Right panel). Top panel is at 500 nm and the bottom panel scale bar is 200 nm.
