# Supplementary material for: Predicting the response of multiple myeloma to the proteasome inhibitor Bortezomib by evaluation of the unfolded protein response
Source: Blood Cancer J. 2016 Jun 10;6(6):e432–. doi: 10.1038/bcj.2016.40 (PMC5141355; doi:10.1038/bcj.2016.40)
Supplement: Supplementary Information [file bcj201640x1.docx]

**Supplementary Information:**

**Method**

**Cell culture:** KMS11 Bortezomib sensitive and resistant cells were provided by Dr Silvia Ling and were cultured using RPMI - 1640 culture medium (Sigma-Aldrich, St. Louis, MO, U.S.A) supplemented with 10% foetal calf serum (FCS) (Gibco, Life Technologies, Victoria, Australia), 5% L-Glutamine (Gibco) and 1% penicillin streptomycin (PenStrep) (Sigma) at 37°C in a 5% CO2 atmosphere. KMS11 Bortezomib resistant cells were cultured in the presence of Bortezomib at 75nM (Velcade, Millennium Pharmaceuticals, Cambridge, MA, U.S.A).

**Live Cell Imaging:** KMS11 Bortezomib sensitive and resistant cells were cultured under previously mentioned conditions in 35 mm glass bottom culture dishes (Greiner Bio-One, Germany) for 24 hours. After 24 hours, the media was aspirated off and replaced with fresh culture media without FCS, and incubated with 100 nM of ER-tracker^TM^ Green (Invitrogen, Life Technologies, Victoria, Australia) at 37°C for 40 minutes. This is a cell permeable stain consistent of a green fluorescent dye and glibenclamide that binds to the sulphonylurea receptors of ATP-sensitive K+ channels, which are prominent on ER. After incubation, media was aspirated and replaced with regular culture media for imaging. Images were captured using the BioStation IM-Q Time Lapse Imaging System (Nikon Corporation, Japan). Image analysis was performed using ImageJ 1.46r software to calculate the corrected total cell fluorescence (CTCF) of Bortezomib sensitive and resistant myeloma cell lines and primary myeloma cells. Mean fluorescent intensity was corrected to the area on interest.

**Collection and purification of multiple myeloma cells:** Forty-five bone marrow samples were collected from MM patients, prior to treatment. All patients were treated with Bortezomib in combination with one or two other drugs. The work was approved by the South Western Sydney Local Health District Human Research Ethics Committee and the Biobank Ethics Committee and Tissue Management Research Committee at the Peter MacCallum Cancer Centre..Bone marrow mononuclear cells were isolated by density centrifugation using Ficoll-Paque Plus (GE Healthcare, Little Chalford, United Kingdom) at 400 g for 35 min at 20°C, washed twice with phosphate buffered saline (PBS) containing 2 mM EDTA (centrifugation at 300 g for 10 min at 20°C) and cryopreserved at -180°C for cell sorting. MM cells from patient samples were isolated by flow cytometry using CD38+ and CD138+ using a two lasers station fluorescence-activated cell sorting system (BD Biosciences, N.S.W, Australia). Prior to sorting, all samples were thawed and washed with 3 ml BPS and centrifuged at 1500 rpm for 5min. 60 µl cells (1x10^5^ - 1x10^6^) was then incubated with 15 µl of CD138-PE (A40316, Beckman Coulter, N.S.W, Australia) and 15 μl of CD 38-APC (340439, BD) on ice (protected from light) for 30 minutes. Following incubation, samples were washed by PBS (3 ml) and centrifuged at 1500 rpm for 5 minutes. For each sample, 15 μl of 7AAD solution (555816, BD) was added into 60 μl cells on ice and incubated for 10 minutes, then washed by PBS (3 ml) twice. Cell pellets were suspended in 500 μl of PBS for cell sorting at the flow cytometry facility of the Westmead Millennium Institute, N.S.W, Australia.

**Clinical Response:** Patient responses were evaluated after the completion of 2 cycles of Bortezomib according to the IMWG uniform response criteria. Briefly, patients were assigned to the complete response (CR) group if they had negative immunofixation IFE on serum and/or urine and less than 5% plasma cells in bone marrow. Patients were in very good partial remission (VGPR) if they were negative on serum electrophoresis but positive on serum IFE or there was a ≥90% reduction of paraprotein in serum plus urinary M component of <100mg/24hour. Partial response (PR) patients had at least a 50% reduction in serum M-protein and a reduction in 24-hour urinary M-protein by 90% or to <200 mg/24 hours. If the serum and urine M-protein are not measurable, PR constitutes a decrease of ≥ 50% in the difference between involved and uninvolved serum free light chain. Progressive disease (PD) patients had an increase of at least 25% of the lowest response value of serum or urine M component, while stable disease (SD) patients didn’t fit the criteria for CR, VGPR, PR or PD.

**Real-Time PCR:** RNA was extracted from cell sorted patient samples and KMS11 cell lines using TRIzol reagent (Invitrogen), followed by RNA clean up using the RNeasy Mini kit (Qiagen, Germany) following the manufactures instructions. RNA was reverse transcribed using SuperScript™ III Reverse Transcriptase (Invitrogen) kit and random hexamer primers (Invitrogen). ATF6 gene expression was quantified on the Rotor-Gene Q (Qiagen), using the standard curve method. ATF6 was normalised to the internal control gene β-actin for each sample, using the following primers; ATF6 Forward: 5’-AATCCGCTTGTCAGTCTCGC-3’ and Reverse: 5’-GCCTCTGGTTCTCTGACACA-3’; β-actin Forward: 5’-AATCTGGCACCACACCTTCTAC-3’ and Reverse: 5’-ATAGCACAGCCTGGATAGCAAC-3’. All samples were run in triplicate and each reaction consisted of 10 ml of KAPA SYBR® FAST qPCR Kit Master Mix (2X) Universal (KAPA Biosystems, Wilmington, MA), 1 μl of forward and reverse primer, 1 μl cDNA, 7 μl of nuclease free water (Geneworks), and amplified over 40 cycles for 95°C (10 sec), 63 °C (15 sec) and 72°C (15 sec), after an initial activation step of 95°C (5 min). A serial dilution of control cDNA was used as the template for each standard curve. The copy number for ATF6 and β-actin was calculated for each sample using the URI Genomics & Sequencing Center copy number formula.

**Electron Microscopy:** Using a Morgagni transmission electron microscope (TEM) (FEI, Eindhoven, The Netherlands), rough endoplasmic reticulum (ER) luminal widths were measured in KMS11 Bortezomib sensitive and resistant cells at a magnification of 40,000x. For each cell type, 10 images of the ER were acquired and the luminal width measured at 4 points (widest points) in each image using online calibrated software (AnalySIS v3.0, Soft Imaging System GmbH, Germany). The mean of the 40 measurements was then calculated for each sample.

**Statistics:** All graphs were generated using GraphPad Prism (GraphPad Software, CA, U.S.A) and statistical analysis was performed by an unpaired *t* test with Welch’s correction student t-test.
